# Evaluation of Candidate Reference Genes for Quantitative Gene Expression Analysis in *Spodoptera exigua* after Long-time Exposure to Cadmium

**DOI:** 10.1038/s41598-017-08630-6

**Published:** 2017-08-21

**Authors:** Anna Płachetka-Bożek, Maria Augustyniak

**Affiliations:** Department of Animal Physiology and Ecotoxicology, University of Silesia in Katowice, Bankowa 9, PL 40-007 Katowice, Poland

## Abstract

Studies on the transcriptional control of gene expression play an important role in many areas of biology. Reference genes, which are often referred to as housekeeping genes, such as GAPDH, G3PDH, EF2, RpL7A, RpL10, TUBα and Actin, have traditionally been assumed to be stably expressed in all conditions, and they are frequently used to normalize mRNA levels between different samples in qPCR analysis. However, it is known that the expression of these genes is influenced by numerous factors, such as experimental conditions. The difference in gene expression underlies a range of biological processes, including development, reproduction and behavior. The aim of this study was to show the problems associated with using reference genes in the qPCR technique, in a study on inbred strains of *Spodoptera exigua* selected toward cadmium resistance. We present and discuss our results and observations, and give some recommendations concerning the use and limitations of housekeeping genes as internal standards, especially in research on insects. Our results suggest that holometabolism and poikilothermia, as well as time since metamorphosis and the level of exposure to the selective factor (cadmium in this case), have a significant effect on the expression of reference genes.

## Introduction

Quantitative real-time PCR is commonly used to measure and evaluate changes in gene expression. For the past 30 years *BioTechniques* has published reports on the use of the qPCR technique in gene expression analyses^1^. Initially, this method was mainly used to measure the level of RNA species through mathematical analysis of qPCR data. The usefulness of qPCR in measuring the levels of mRNA species, as a more specific reverse-transcription quantitative PCR (RT-qPCR), was examined several times. This technique was developed and became widely used in modern biology and biomedical sciences, and it has progressed in tandem with the microarray field. Until today qPCR (despite its limitations) is a basic method used for gene expression analysis, but microarrays are still prefered if the analysis involves a very large number of samples. However qPCR is still a rapid and easy method to validate microarrays results^1^. Currently, this technique is suitable for many applications, e.g. gene expression analysis, single nucleotide polymorphism (SNP) genotyping, miRNA analysis, copy number variation (CNV) analysis, etc. Examination of gene expression levels is very important for identification of genes that participate in a variety of biological processes and provide necessary data to complex regulatory networks^2^. The qPCR technique is very sensitive and can detect changes for a very low transcript level. This is both a strong point and a weakness of this method. Due to its high sensitivity, this method is subject to significant errors, and it is associated with different amounts of starting material, the quality and integrity of the mRNA, RT-PCR and qPCR efficiency and differences in the transcriptional activity of analyzed tissues^3^. When all of these conditions are met, the next requirement for a reliable qPCR assay is to check the expression level and stability of reference genes (so-called housekeeping genes), because they are used as internal controls for normalizing gene expression. By definition, the expression level of reference genes should be stable across the different treatments and/or tissue types in an experiment^4^.

The perfect reference genes should be expressed in abundance and have minimal innate variability^2^. Several studies have shown that this approach can introduce large errors, especially when the expression of the reference genes is measured for different treatments and/or in different tissues^2^. The mRNA level of housekeeping genes can depend on numerous factors, such as the stage of an organism’s development, cell cycle, experimental conditions^5^, time of day when the material was collected (often associated with photoperiod), etc. The difference in gene expression underlies a range of biological processes, including development, reproduction and behavior^6^.

Almost ten years ago, Verma and Shapiro (2006) discovered and described sex-dependent expression of several genes that are commonly used as reference genes in real-time PCR^7^. This team reported constitutive sexual dimorphism of hepatic mRNA level of seven commonly measured housekeeping genes such as tubulin, glyceraldehyde 3-phosphate dehydrogenase, β-actin and ribosomal protein 18S in rat liver. This is probably connected with varying hormonal regulation, although the particular hormone(s) involved have not been identified^7^. This result shows that different factors (even normal physiological conditions) can influence the expression of different genes, including housekeeping genes. In the last decade, when performing various mRNA quantification experiments, most scientists observed that expression levels of housekeeping genes that are used as internal standards can fluctuate^5^. Today, it is commonly known that many reference genes should be experimentally validated for specific experimental designs. In some situations, even normalization against several reference genes can be insufficient^8^. Nevertheless, most researchers were routinely assuming housekeeping gene expression levels to be constant without discussing the issue^5^. In this work, we would like to present and discuss our results and observations, as well as give some recommendations concerning the use and limitations of housekeeping genes as internal standards, especially in research on insects. Two inbred strains (cadmium and control) of *Spodoptera exigua* were used and expression of candidate reference genes such as: glyceraldehyde 3-phosphate dehydrogenase (GAPDH), glycerol-3-phosphate dehydrogenase (G3PDH), elongation factor 2 (EF2), ribosomal protein L7A (RpL7A), ribosomal protein L10 (RpL10), alpha tubulin (TUBα) and cytoplasmic actin gene (ACT) was measured in the fat body (FB) at many time points after eclosion. Therefore, it was possible to assess the usability of a wide array of reference genes in research done on insects after complete metamorphosis.

## Results

The stability of the candidate reference genes was analyzed using geNorm, NormFinder, BestKeeper and basic statistical methods (median, min–max) to determine crossing point-PCR-cycle value (Cp value) dispersion.

### Analysis of the crossing point dispersion

**Figure 1:**
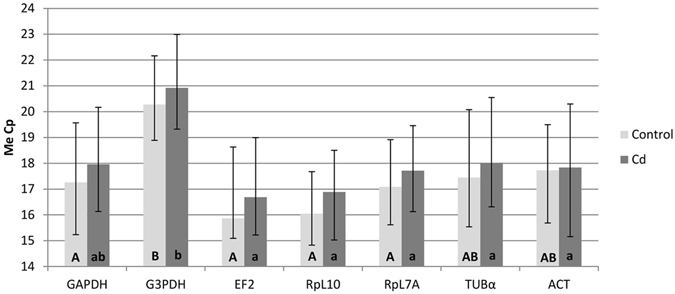
Results of the gene expression analysis. Medians (Me) of Cp (with the min/max) were calculated for all of the individuals from all of the experimental time groups. Statistical differences between genes within experimental group (control and cadmium) were calculated using the Kruskal-Wallis test (p < 0.05). Statistical differences within one gene between experimental groups (control and cadmium) were calculated using U Mann-Whitney test (p < 0.05). Abbreviations: glyceraldehyde 3-phosphate dehydrogenase (GAPDH), glycerol-3-phosphate dehydrogenase (G3PDH), elongation factor 2 (EF2), ribosomal protein L7A (RpL7A), ribosomal protein L10 (RpL10), alpha tubulin (TUBα) and cytoplasmic actin gene (ACT). The same capital letters means: no significant differences between genes within control strain. The same small letters means: no significant differences between genes within cadmium strain.

**Figure 2:**
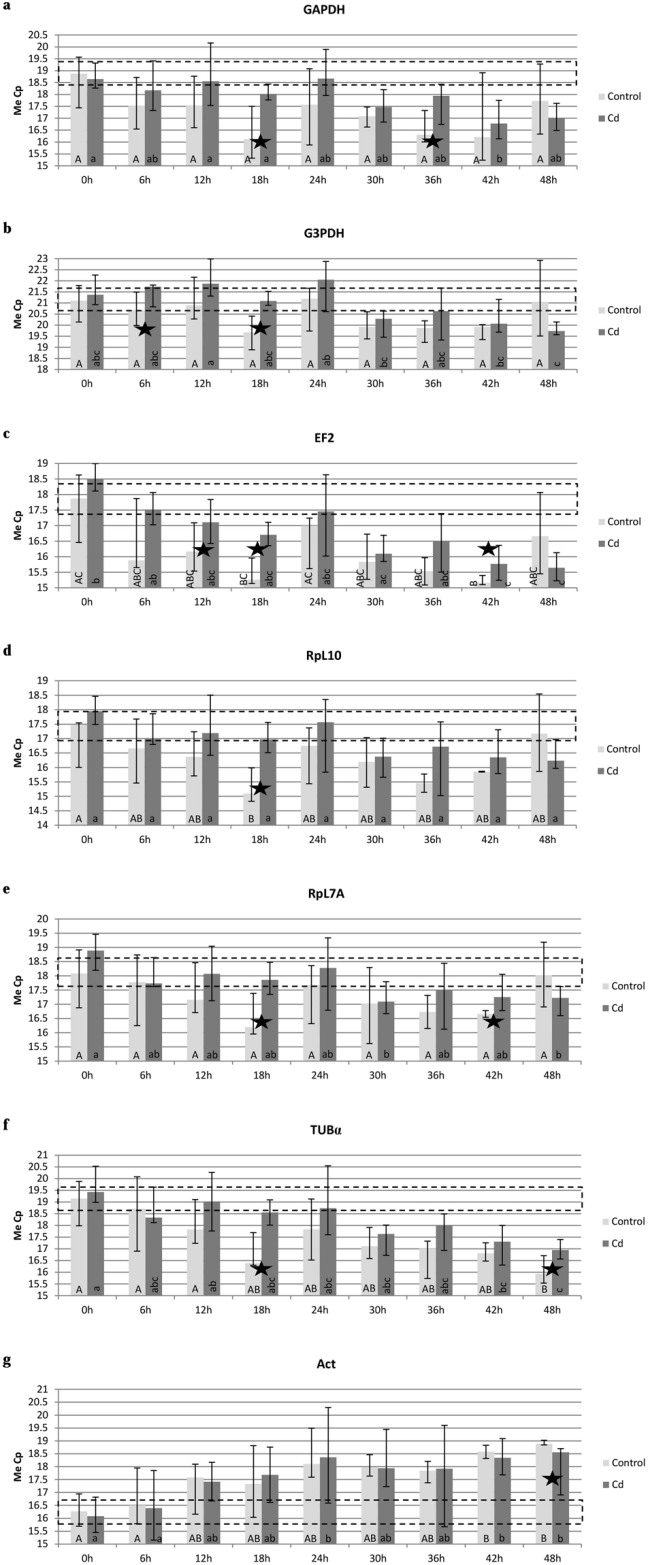
Comparison of the housekeeping genes expression in the control and cadmium strain of S*podoptera exigua* at different time points for: (**a**) GAPDH, (**b**) G3PDH, (**c**) EF2, (**d**) RpL7A, (**e**) RpL10, (**f**) TUBα and (**g**) ACT. Statistical differences between time-potins within experimental group (control and cadmium) were calculated using the Kruskal-Wallis test (p <  0.05) and between experimental groups (control and cadmium) within one time-point were calculated using U Mann-Whitney test (p < 0.05). ★Differences between breeding strains: control and cadmium.  Acceptable median of Cp value with its min-max value. Abbreviations: glyceraldehyde 3-phosphate dehydrogenase (GAPDH), glycerol-3-phosphate dehydrogenase (G3PDH), elongation factor 2 (EF2), ribosomal protein L7A (RpL7A), ribosomal protein L10 (RpL10), alpha tubulin (TUBα) and cytoplasmic actin gene (ACT). The same capital letters means: no significant differences among time groups within control strain. The same small letters means: no significant differences among time groups within cadmium strain.

In these studies the crossing point dispersion was analyzed first. Figure 1 shows Cp values and gives an overview of variation in gene expression within and between the experimental groups. The largest dispersion (for all samples) of Cp was identified for the ACT gene at 5.14 cycle for all of the samples (Table S2). For this gene, the variation of Cp value was different compared to the experimental groups: the minimal variation of Cp value was 0.18 cycle in the control strain 48 hrs after eclosion, and this variation was 1.80 cycle in the cadmium strain, however maximal variation was observed in the cadmium strain 36 hrs after eclosion (it was 3.94 cycle) while at the same time point in the control strain this value was 0.82 cycle (Fig. 2g and Table S1).

For all samples, the smallest dispersion of Cp was noted for the RpL10 gene and it was 3.72 cycle (Table S2). Between the experimental groups, minimal variation (0.03 cycle) of Cp value was visible 42 hrs after eclosion in the control strain, but this variation was 1.52 cycle in the cadmium strain. Maximal variation of Cp value (2.68 cycle) was identified 48 h after eclosion in the control strain, while variation of Cp value was much smaller in the cadmium strain at this time-point (1.0 cycle) (Fig. 2d and Table S1). The presented data shows that expression levels of the examined genes were regulated differently in both control and cadmium strains. The difference in expression level of “housekeeping genes” between the strains at each analyzed time-point was surprising, because the difference exceeded two cycles (Fig. 2a–g, Tables S1 and S2).

In this study two tendencies can be observed – the GAPDH, G3PDH, EF2,RpL10, RpL7A genes showed a higher expression at 0 h (except GAPDH), 6 h, 12 h, 18 h, 24 h, 30 h, 36 h and 42 h in individuals from the cadmium strain compared to the control. At 48 h the difference of expression was opposite. The expression of TUBα gene shows decreasing tendency over time in both breeding strains, and statistical differences were demonstrated between first and last time-points (Fig. 2f and Table S1). However, actin gene showed a reverse dependency (Fig. 2g). General, cyclic variations in gene expression were observed in both strains, but in the control group this dependence is more evident. Visible changes of gene expression for GAPDH, G3PDH, EF2, RpL10, RpL7A and TUBα have daily dependence in the control strain: a decrease from 0 h to 18 h, an increase at 24 h, another decrease from 24 h to 42 h and again an increase at 48 h (except TUBα). Although these dependences were not observed in the cadmium strain, 24 hours after eclosion, the expression of these genes is decreased (Fig. 2a–g and Table S1). The TUBα showed a decrease in expression in both strains and ACT showed an increase in expression over time (Fig. 2f,g, Table S1).

### Attempt to identify the best reference gene for qPCR

**Figure 3:**
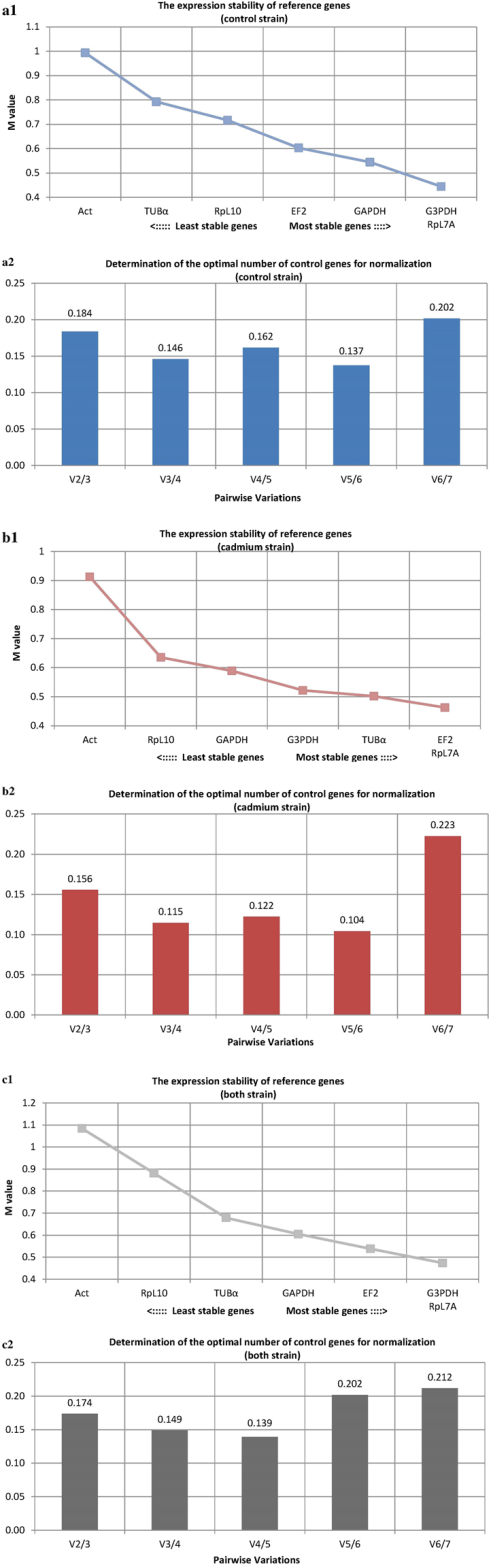
Expression stability of 7 candidate reference genes as calculated by geNorm software to check expression stability (**a1,b1,c1**) and indicate the optimum number of reference genes required for qPCR data normalization (**a2,b2,c2**). Abbreviations: (a) control strain, (b) cadmium strain, (c) both strain (all samples), GAPDH – glyceraldehyde 3-phosphate dehydrogenase, G3PDH – glycerol-3-phosphate dehydrogenase, EF2 – elongation factor 2, RpL7A – ribosomal protein L7A, RpL10 – ribosomal protein L10, TUBα – alpha tubulin, ACT – cytoplasmic actin gene.

**Figure 4:**
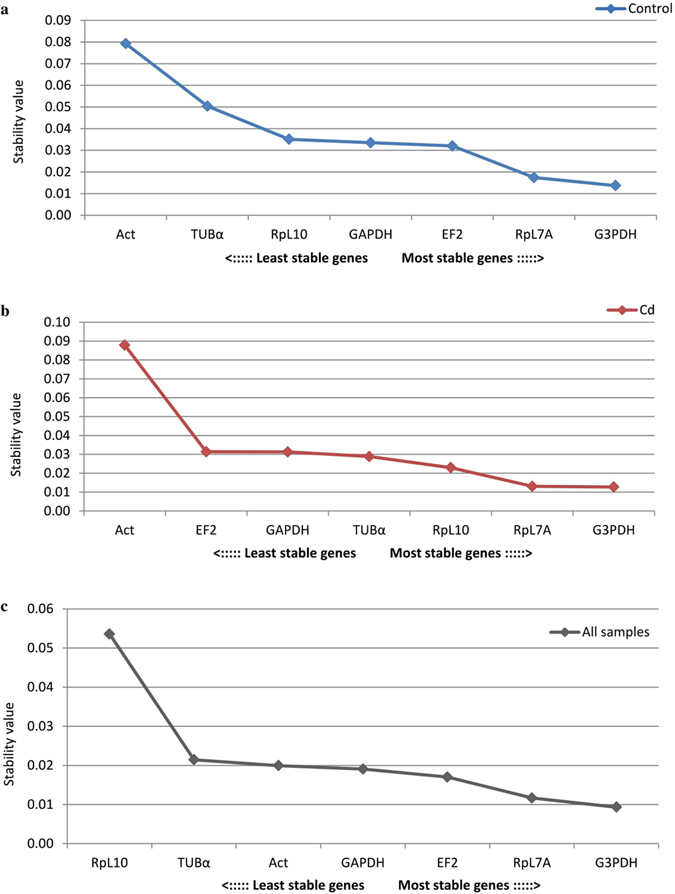
Expression stability of 7 candidate reference genes as calculated by NormFinder. Abbreviations as in Fig. 3.

**Figure 5:**
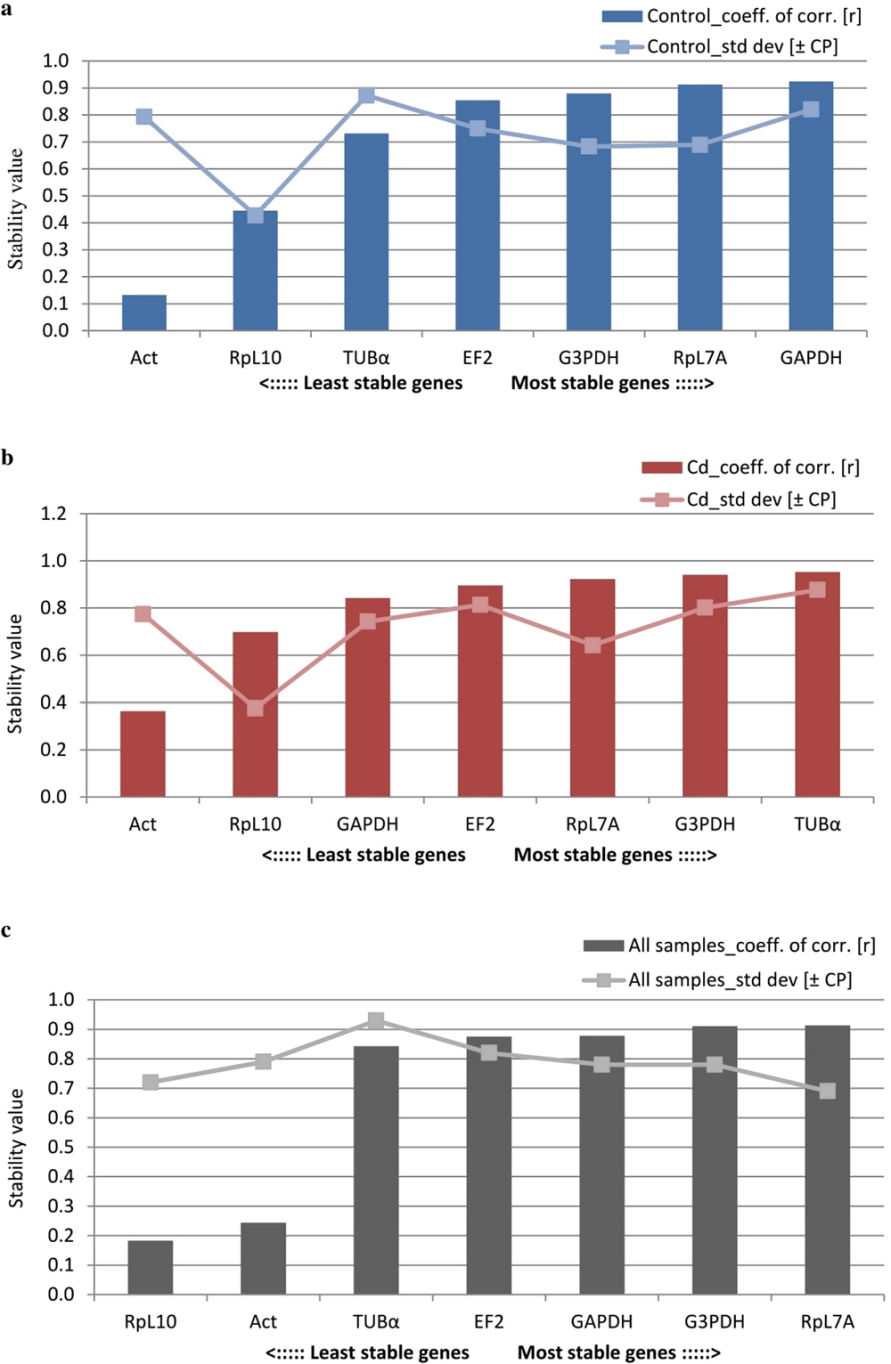
Expression stability of 7 candidate reference genes as calculated by BestKeeper. Abbreviations as in Fig. 3.

**Figure 6:**
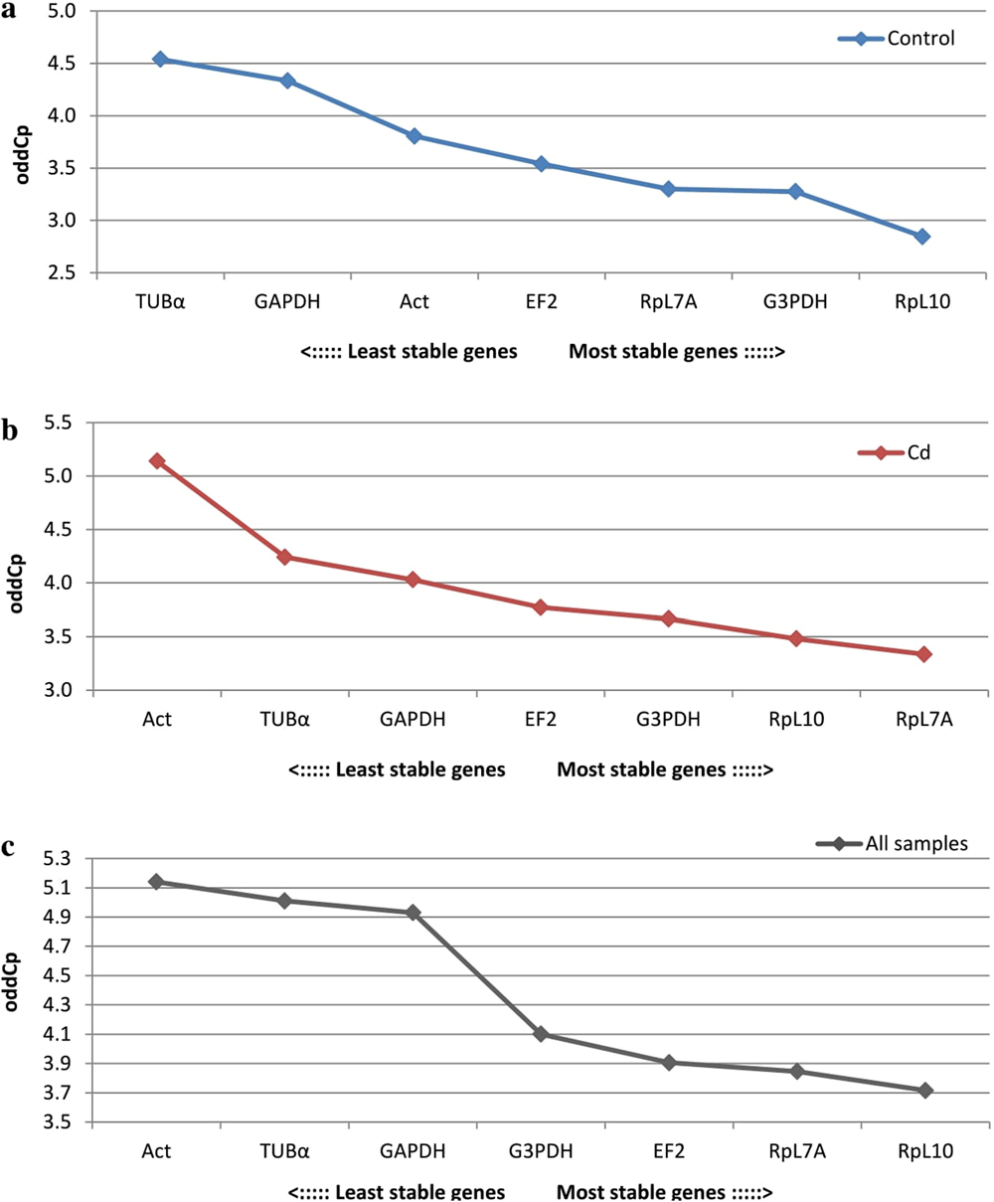
Expression stability of 7 candidate reference genes calculated by differences of crossing point-PCR-cycle values (oddCp = Cp_max_ − Cp_min_). Abbreviations as in Fig. 3.

**Figure 7:**
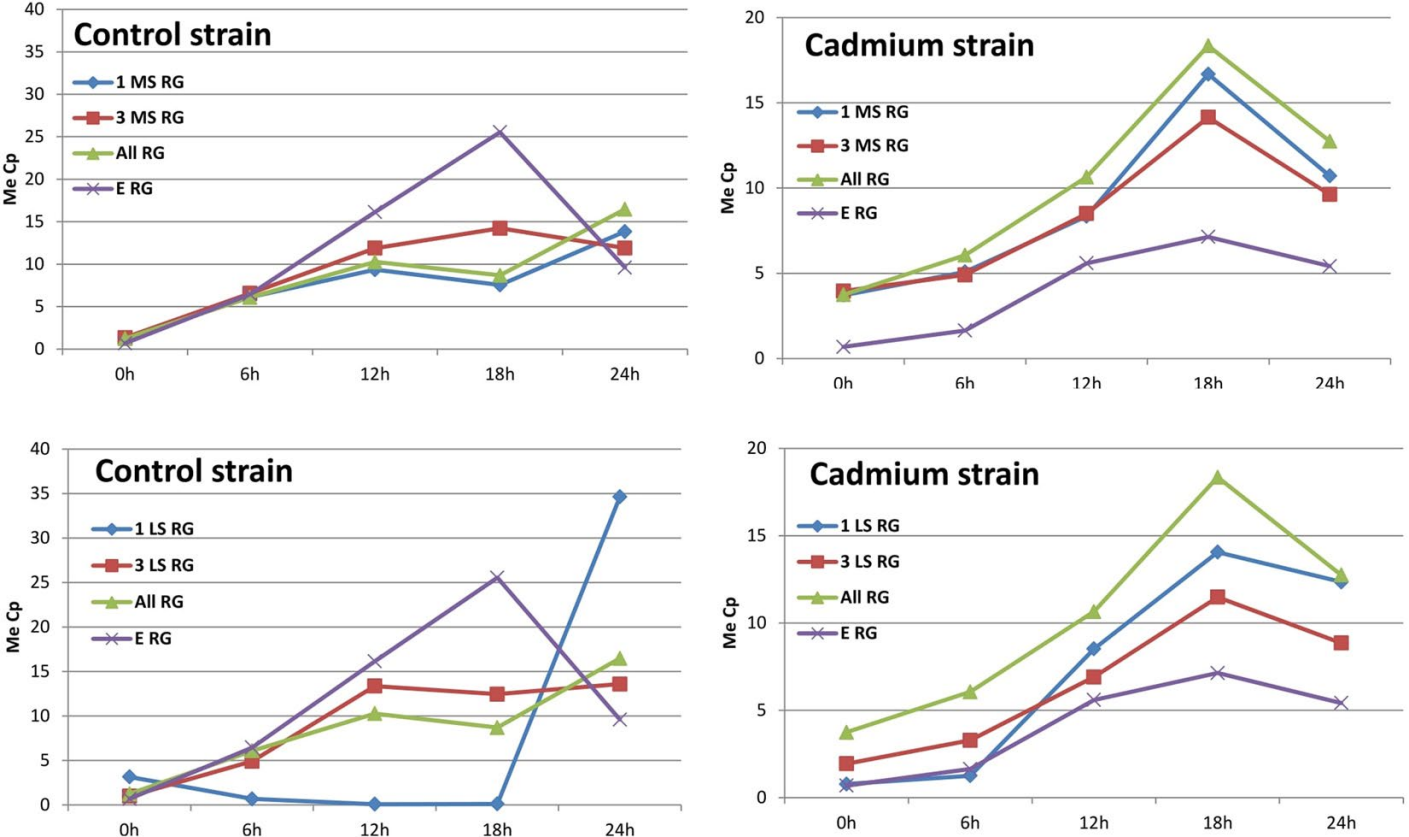
The daily pattern of the vitellogenin gene expression in two breeding strains of *Spodoptera exigua* (control and cadmium) by using: three most stable (3 MS RG), three least stable internal reference genes (3 LS RG), one most stable internal reference gene (1 MS RG), one least stable internal reference gene (1 LS RG) and external reference gene (E RG) and all reference genes (All RG) in qPCR analysis (according to the general ranking shown in the Table 1). Abbreviations: 0 h, 6 h, 12 h, 18 h, 24 h - time after eclosion.

The smallest deviations for all samples was observed for the RpL10 gene, as mentioned above, but commonly used algorithms to determine gene expression stabilities such as: geNorm, NormFinder, BestKeeper and crossing point-PCR-cycle value dispersion (oddCp) yielded different results (Figs 3–6). After analyzing all the data, the best gene to use as a reference was RpL7A, followed by G3PDH and EF2 (Table 1). In the first case the difference was 3.85 cycles, with all samples analyzed (Fig. 1 and Table S2). Additionally at 18 and 42 hours expression level was statistically lower in the control strain compared with the cadmium strain (Fig. 2e). Differences between time-points were also observed within the cadmium strain: at 30 and 48 hours the expression level was lower than at 0 hour after eclosion. This same problem applied to the other two preferred genes (G3PDH and EF2). For G3PDH differences between experimental groups occurred at 6 and 18 hours and within cadmium strain there were differences between time-points (Fig. 2b), while for EF2 genes these differences between and within experimental groups were bigger (Fig. 2c). It is important to notice that the smallest statistical differences between and within experimental groups were observed for RpL10 and ACT genes. However, according to used algorithms, actin gene has the lowest stability of expression and RpL10 gene ranks third last (Table 1). Analysis of determination of the optimal number of control genes for normalization show that in this experiment the three most stable reference genes should be used to obtain the best results of target gene expression analysis (Fig. 3: a2, b2, c2). In this case all of these genes demonstrated daily variability (Fig. 2a–g) and it would be a mistake to use them to determinate expression of a target gene, the expression of which varies during the day. Moreover these genes display a difference between breeding strains (control and cadmium) which creates an additional complication in the target gene expression analysis. The differences in expression level in candidate of reference genes and the observed diurnal rhythms excludes their use for determination of expression levels in genes like the vitellogenin gene (Fig. 7). The differences between strains and the diurnal rhythms flatten the results of gene expression.

**Table 1:** Expression stability ranking of the 7 candidate reference genes.

**Method**	**1**	**2**	**3**	**4**	**5**	**6**	**7**
**Ranking order under control samples (most stable genes ->least stable genes)**							
**geNorm**	RpL7A	G3PDH	GAPDH	EF2	RpL10	TUBα	Act
**NormFinder**	G3PDH	RpL7A	EF2	GAPDH	RpL10	TUBα	Act
**BestKeeper**	GAPDH	RpL7A	G3PDH	EF2	TUBα	RpL10	Act
**oddCp**	RpL10	G3PDH	RpL7A	EF2	Act	GAPDH	TUBα
**General ranking**	**RpL7A**	**G3PDH**	**GAPDH**	**EF2**	**RpL10**	**TUBα**	**Act**
**Ranking order under cadmium samples (most stable genes ->least stable genes)**							
**geNorm**	RpL7A	EF2	TUBα	G3PDH	GAPDH	RpL10	Act
**NormFinder**	G3PDH	RpL7A	RpL10	TUBα	GAPDH	EF2	Act
**BestKeeper**	TUBα	G3PDH	RpL7A	EF2	GAPDH	RpL10	Act
**oddCp**	RpL7A	Rp10	G3PDH	EF2	GAPDH	TUBα	Act
**General ranking**	**RpL7A**	**G3PDH**	**TUBα**	**EF2**	**RpL10**	**GAPDH**	**Act**
**Ranking order under total samples (most stable genes ->least stable genes)**							
**geNorm**	RpL7A	G3PDH	EF2	GAPDH	TUBα	RpL10	Act
**NormFinder**	G3PDH	RpL7A	EF2	GAPDH	Act	TUBα	RpL10
**BestKeeper**	RpL7A	G3PDH	GAPDH	EF2	TUBα	Act	RpL10
**oddCp**	RpL10	RpL7A	EF2	G3PDH	GAPDH	TUBα	Act
**General ranking**	**RpL7A**	**G3PDH**	**EF2**	**GAPDH**	**RpL10**	**TUBα**	**Act**

## Discussion

HKG is used in normalization of real-time quantitative RT-PCR data due to the assumption that expression levels of these genes remain unchanged. Due to numerous environmental factors, sex, age, etc. influencing cell metabolism, life processes that are responsible for homeostasis fluctuate. Therefore, stability of basic metabolism genes has often been questioned^7–9^. Many studies have shown that each program has its own algorithm to examine stable gene expression patterns and each of them can show different results^10, 11^. To evaluate the gene expression stability of the selected genes among different study groups, scientists use programs such as geNorm^12^, BestKeeper^13^, NormFinder^3^ or crossing point-PCR-cycle value comparison. GeNorm uses pair-wise variation of each of the genes as standard deviation and calculates their expression values and their stability^2^. BestKeeper calculates pair-wise correlation and compares it to each individual to obtain an index based on the geometric mean of the selected HKG^5^. The NormFinder algorithm estimates expression stabilities according to intra- and inter-group variations, and crossing point-PCR-cycle value is based on simple comparison of Cp value between samples and experimental groups^10^. For our study, the algorithms are not useful to examine candidate genes’ expression stability. There is information suggesting that each of these algorithms gives different results depending on the experimental group^2, 10, 14, 15^. The gene expression normalization factor cannot be calculated based on the geometric mean of a user-defined number of reference genes. For these studies, it was important to check the distribution of differences between samples. The minimum and maximum parameters showed clear instability in housekeeping gene expression. Usage of min-max parameters showed the real variation between every single sample (Figs 1–2). Many researches and large biotechnology companies accept at most a 0.5 cycle difference between samples and believe that it is acceptable to use a gene as a reference for qPCR analysis. In this experiment, the odds between the min Cp and max Cp was 3.72 cycle for the RpL10 gene. This was the smallest variation between the min Cp and max Cp among the analyzed genes. Because of the above-mentioned criteria, this result unfortunately does not allow any of the tested genes to be used as a reference.

The factors that influence HKG expression are assumed to be numerous. The position of a gene in a genome, its sequence and function in a given tissue seem to be significant. Other than the typical genetic factors, the influence of certain paragenetic and exogenous factors should also be considered in context of reference gene stability^16^.Sex, along with the resulting hormone levels and age of an individual, all influence the HKG expression level. It has been known for some time that reference gene expression is changed in tumor cells. For example, GAPDH expression has been proven to be higher in tumor cells (e.g. pancreas, stomach, colon and liver tissues) compared to healthy tissues^17^. The expression level of two housekeeping genes β-actin and RpL30 (ribosomal protein) was checked by researchers from Austria and they proved that expression of these genes were dramatically reduced in human peripheral blood mononuclear cells after exposure to concentrations of 100 μmol/l of cadmium. They suggested that expression of housekeeping genes as a reference can only be used under noncytotoxic experimental conditions^18^. The difficulties of interpretation of qPCR results were also noticed by the team from the Washington State University. The researchers emphasized the lack of a suitable reference gene due to the dynamic nature of endogenous transcription and suggested to use an exogenous mRNA as an alternative for analysis of qPCR data^19^. Another team reports that the expression level of different housekeeping genes in *Lipaphis erysimi* is unstable especially when analyzing more factors together: developmental stage, temperature or artificial diet. In this case the pair-wise variation values were above the cut-off value^20^.

The matter becomes even more complicated for poikilothermic organisms, the life cycles of which involve significant periodic changes of tissues and organs. This is the case for insects, especially species that undergo a complete metamorphosis. Their life cycle includes larvae, pupae and adults, all with different behaviors, varied food preferences (often radically different) and changing ecological niches (leading to different environmental stressors). Poikilothermia is also characteristic for drastic changes in metabolism rate^21–23^. In the physiology of insects, which remain under strong environmental stress, the most efficient strategy is to use reserve energy in the most economical way for the needs and capabilities of a particular organism^21, 24^. This aspect of the life of poikilotherms should be taken into account when designing qPCR experiments and analyses, and selecting sets of reference genes.

In our research, which was conducted on two inbred strains of *S. exigua*, we proved that transcription levels were different in all seven of the reference genes that were analyzed at each time-point within and between control and cadmium strains (Figs 1–2). These results allow us to state that poikilothermia, time since metamorphosis and level of exposure to the selective factor (cadmium in this case) all have a significant effect on expression of reference genes. This creates a serious problem when using these genes for normalization of quantitative PCR in research on insects. Nonetheless, research on this group of animals using qPCR has invaluable benefits. Therefore, the issue of variability in gene expression between samples and experimental groups should still be discussed so that the most useful solution can become the standard in research on insect material. Our research shows (Fig. 7) that using an internal reference gene (genes) can give false and flattened results. We examined the daily timing of vitellogenin gene expression in insects and observed that using the first and the third most stable internal reference genes and external reference gene gave us different results as shown in Fig. 7. The results obtained using external reference genes are in accordance with published data^25, 26^, and only in this case it is possible to observe gene expression pattern characteristic for vitellogenin (Fig. 7).

Our suggestion for solving this problem is to use an external reference gene in research on insect tissues. The gene should have a starting matrix density that is strictly defined for any given analysis. We know that the expression level of the genes that are commonly used as a reference in the fat body of insects can be up- or down-regulated, especially under various types of stimulation^9^. The potential use of an external reference gene can be a good solution, although it does have some restrictions. The isolation of RNA in each of analyzed samples must be perfectly conducted. After isolation, the samples should be checked on gel and should be perfectly and equally diluted. In this study, each of the samples was checked on RNA 6000 Nano Chip. The worst samples (2, 3 and 6 – Fig. S1) were rejected from further analysis. The fragment of the selected reference gene in a plasmid can give us a stable point to use the ∆∆Cp method to examine the target genes’ expression.

## Conclusions

In summary, this is the first report of housekeeping genes’ expression in two inbred strains of *Spodoptera exigua* – control and long-term selected towards cadmium resistance. Detailed analysis of housekeeping genes expression during the day showed significant differences in the expression of those genes, both within control and cadmium breeding strains, and between control and cadmium strains. These results allow us to state that cadmium has a significant effect on the expression of housekeeping genes. This creates a problem when using these genes for the normalization of quantitative PCR in research on insects.

## Material and Methods

### Insects

The Department of Animal Physiology and Ecotoxicology at the University of Silesia in Katowice conducts a breeding program of *Spodoptera exigua*. One strain of these insects has been fed a diet contaminated with cadmium (44 mg Cd/kg dry weight of larval diet) for 120 generations. The second strain of insects (control) was reared on a standard artificial diet for insects. Individuals from both strains were kept in the following conditions: at 25 ± 1 °C, a photoperiod 16 L:8 D (light:dark) and RH 30 ± 5%.

### Sample collection, tissue preparation and homogenization

The pupae were separated by sex, kept in 24-well plates and registered every 30 minutes using a Life Web Camera connected to a computer with Timelapse software (available online) and a screen emitting red light. Insects were frozen in liquid nitrogen at the following time points after adult eclosion: 0 h, 6 h, 12 h, 18 h, 24 h, 30 h, 36 h and 48 h. It was analyzed from 7 to 10 individuals for each strain at each time point.

Before RNA isolation, the fat body (FB) was separated from each individual and treated with an RNAlater Storage Solution (Sigma-Aldrich, Germany). Tissues from each individual were homogenized in 250 µL of QIAzol Lysis Reagent (Qiagen, Germany) using FastPrep® Systems (MP Biomedicals, USA).

According to qPCR publications, seven commonly used candidate reference genes, in this case housekeeping genes, were selected to validate their expression stability (Table 1): GAPDH, G3PDH, EF2, RpL7A, RpL10, TUBα and ACT. Primers were tested by melt curves and product size on a gel analysis. The fat body was chosen for these studies because it is a dynamic tissue that is involved in multiple metabolic functions that are fundamental in the life of holometabolous insects such as Lepidoptera^24^. In context of the study on stability of housekeeping gene expression, they take on a special meaning.

### Total RNA isolation and cDNA synthesis

After treatment with QIAzol Lysis Reagent, samples were incubated for 5 min at room temperature and 200 µL chloroform was added. Tubes were shaken vigorously for 15s, incubated for 3 min and centrifuged for 15 min at 12,000 × *g* at 4 °C. The upper aqueous phase was transferred to a new 1.5 ml RNase-free tube. 120 µL isopropanol was added and samples were incubated at RT for 10 min and vortexed for 5s. Next, samples were spun at max *g* at 4 °C for 15 min. The supernatant was removed and the pellet was washed in cold 70% EtOH, spun max *g* for 10 min at 4 °C and air-dried for 10 min. Total RNA was eluted in 30 µL of nuclease free water. Total RNA was treated with a DNase I recombinant, RNase-free (Roche, Switzerland), incubated with enzyme at 37 °C for 1 h, and then with 3 M sodium acetate and 96% EtOH at −70 °C overnight. Samples were spun at max *g* at 4 °C for 20 min, the supernatant was removed and the pellet was washed in cold 96% and 70% EtOH. Total RNA was eluted in 20 µL of nuclease free water. RNA was stored at −70 °C before further processing. The quality and quantity of RNA was examined using a NanoDrop-2000 (Thermo Fisher, USA) and 2100 Bioanalyzer Instrument with Agilent RNA 6000 Nano Kit (Agilent Technologies, USA) (Fig. S1). cDNA was synthesized using a Maxima First Strand cDNA Synthesis Kit (ThermoFisher Scientific, USA) and was stored at −20 °C until use.

### Plasmid DNA isolation

In real-time qPCR external reference gene has also been used. It was actin gene cloned into plasmid – pMT-EGFP-Actin5c, it was a gift from Ron Vale (Addgene plasmid #15312)^27^. DNA from bacteria was isolated by using FastGene Plasmid Mini Kit (Nippon Genetics, Japan). The purity and quality of the DNA was checked by electrophoresis on agarose gel and DNA concentration was estimated by measuring the absorbance at 260 nm by using NanoDrop 2000 (Thermo Fisher, USA).

### Quantitative real-time PCR

Based on literature^2, 14^ and known insect RNA sequences of the ACT, GAPDH, G3PDH, EF2, RpL10, RpL7A and TUBα genes (Table S3, Fig. S2), the ACT primers were designed with the Pirimer3 software that is available online (http://bioinfo.ut.ee/primer3-0.4.0/) and the others were used from the literature and they were checked using the NCBI Database before the experiments. The 2% agarose gel electrophoresis was used to check PCR product (to confirm the specificity and the absence of primer dimer formation, Fig. S2). Gene specific amplification was also confirmed by a single peak in melt-curve analysis. For each primer, reaction efficiency was determined by a standard curve of cDNA samples (Table S3) according to the MIQE guidelines for qPCR^28^.

Duplicate first strand cDNA aliquots for each sample served as the templates for qPCR using SYBR Green I (Roche, Switzerland) on a LightCycler 480 (Roche, Switzerland). Amplification reactions were performed in 10 µL total volumes with 20 ng cDNA, 0.25 pmol each of primers, 5 µL SYBR Green in 96-well white plates (Roche, Switzerland) under the following thermal conditions: 95 °C for 5 min and 40 cycles; 95 °C for 10s, 58 °C for 30s, 72 °C for 30s. The efficiency of a reaction was determined for each gene with the slope of the linear regression model^29^. Standard curves for the gene transcripts were generated with serial dilutions of cDNA: 40 ng, 20 ng, 10 ng, 5 ng, 2.5 ng, 1.25 ng, 0.625 ng per reaction. The stock cDNA used for the relative standard curves was synthesized based on mix RNA from the individuals of the control group six hours after adult eclosion. Expression levels were determined as the number of PCR cycles until the fluorescence intensity of the products exceeded the background (Cp). The threshold was set automatically for all of the genes and the corresponding Cp values were transformed into quantities via the standard curve using the efficiency of the PCR. The samples were run in triplicate and later due to the good reproducibility in duplicate. Pooled RNA samples (transcribed to cDNA), from 10 individuals from control strain (right after eclosion) was used as calibrator for normalized target gene (vitellogenin in this case) expression data. To ensure quality control, both reference cDNA and NTC (no template controls) were included in each run. The negative control was performed in RT-PCR and qPCR. Additionally for all tested genes total RNA as template was used to check total digestion of genomic DNA after nucleic acid isolation.

### Data mining and statistical analysis

The stability of the candidate reference genes was ranked by using Microsoft Excel 2010 based software tools and they were analyzed by using geNorm, NormFinder, BestKeeper algorithm and Cp value comparison. GeNorm algorithm calculates an expression stability value (M) and ranks genes in an order for a given set of samples. The stability value (M value) of less than 1.5 recommended the gene for use as a reference and pair-wise variation value (V) between two sequential normalization factors is used to determine the optimal number of reference genes required for better normalization (a threshold value below 0.15 is required)^12, 20^. NormFinder calculates gene expression stability for all the samples based on intra- and inter-group variations. In addition, the algorithm provides a gene rank order depending on variation in gene expression. The most stably expressed gene/genes have the lowest rank and is/are ideal to select as reference gene(s) for that particular experimental conditions^3, 20^. BestKeeper uses Cp values and PCR efficiency to determine the best suited standards, and combines them into an index^13^. Crossing point-PCR-cycle value (Cp value) is a direct result obtained from the thermal cycler for each of the samples. The Cp value was used to analyze the comparison of gene expression measurement between samples and experimental groups.

Differences between time-points within experimental groups were calculated using Krusk all-Wallis test and differences between experimental gropus within time-points were calculated using U Mann-Whitney test (p< 0.05).

## Electronic supplementary material

Supplementary material

## References

[1] VanGuilder HD, Vrana KE, Freeman WM (2008). Twenty-five years of quantitative PCR for gene expression analysis. Biotechniques.

[2] Zhu X (2014). Selection and evaluation of reference genes for expression analysis using qRT-PCR in the beet armyworm Spodoptera exigua (Hübner) (Lepidoptera: Noctuidae). PLoS One.

[3] Andersen CL, Jensen JL, Orntoft TF (2004). NormFinder\rNormalization of real-time quantitative reverse transcription-PCR data: a model-based variance estimation approach to identify genes suited for normalization, applied to bladder and colon cancer data sets. Cancer Res.

[4] Ponton F, Chapuis M-P, Pernice M, Sword GA, Simpson SJ (2011). Evaluation of potential reference genes for reverse transcription-qPCR studies of physiological responses in Drosophila melanogaster. J. Insect Physiol..

[5] Thellin O, ElMoualij B, Heinen E, Zorzi W (2009). A decade of improvements in quantification of gene expression and internal standard selection. Biotechnol. Adv..

[6] Harshman LG, James AA (1998). Differential gene expression in insects: transcriptional control. Annu. Rev. Entomol..

[7] Verma AS, Shapiro BH (2006). Sex-dependent expression of seven housekeeping genes in rat liver. J. Gastroenterol. Hepatol..

[8] Kozera B, Rapacz M (2013). Reference genes in real-time PCR. J. Appl. Genet..

[9] Peng R (2012). Analysis of reference gene expression for real-time PCR based on relative quantitation and dual spike-in strategy in the silkworm Bombyx mori. Acta Biochim. Biophys. Sin. (Shanghai)..

[10] Cardoso GA, Matiolli CC, de Azeredo-Espin AML, Torres T (2014). T. eixeira. Selection and validation of reference genes for functional studies in the Calliphoridae family. J. Insect Sci..

[11] Zhang, Y. *et al*. Selection of suitable reference genes for quantitative real-time PCR gene expression analysis in Salix matsudana under different abiotic stresses. *Sci. Rep. 7*, 40290; doi:10.1038/srep40290 (2017)*.* 10.1038/srep40290 PMC5264508 28120870

[12] Vandesompele J (2002). Accurate normalization of real-time quantitative RT-PCR data by geometric averaging of multiple internal control genes. Genome Biol..

[13] Pfaffl, M. Quantification strategies in real-time PCR. In *A-Z of quantitative PCR* (ed. Bustin, S. A.) 87–112 (2004).

[14] Teng X, Zhang Z, He G, Yang L, Li F (2012). Validation of reference genes for quantitative expression analysis by real-time rt-PCR in four lepidopteran insects. J. Insect Sci..

[15] Galeano E, Vasconcelos TS, Ramiro DA, De Martin VDF, Carrer H (2014). Identification and validation of quantitative real-time reverse transcription PCR reference genes for gene expression analysis in teak (Tectona grandis L.f.). BMC Res. Notes.

[16] Shen AG, Huang Y, Jiang X, Dou W (2013). Effect of β -Cypermethrin Exposure on the Stability of Nine Housekeeping Genes in Bactrocera dorsalis (Diptera: Tephritidae). BioOne.

[17] Rubie C (2005). Housekeeping gene variability in normal and cancerous colorectal, pancreatic, esophageal, gastric and hepatic tissues. Mol. Cell. Probes.

[18] Marth E, Jelovcan S, Kleinhappl B, Gutschi a, Barth S (2001). The effect of heavy metals on the immune system at low concentrations. Int. J. Occup. Med. Environ. Health.

[19] Johnston S, Gallaher Z, Czaja K (2012). Exogenous reference gene normalization for realtime reverse transcriptionpolymerase chain reaction analysis under dynamic endogenous transcription. Neural Regen Res.

[20] Koramutla MK, Aminedi R, Bhattacharya R (2016). Comprehensive evaluation of candidate reference genes for qRT-PCR studies of gene expression in mustard aphid, Lipaphis erysimi (Kalt). Sci. Rep..

[21] Neven LG (2000). Physiological responses of insects to heat. Postharvest Biol. Technol..

[22] Makarieva AM, Gorshkov VG, Li B (2005). Gigantism, temperature and metabolic rate in terrestrial poikilotherms. Proc. Biol. Sci..

[23] Jaworski T, Hilszczański J (2013). The The effect of temperature and humidity changes on insects development their impact effect of temperature and humidity changes on insects development and their impact on forest ecosystems in the context of expected climate change on forest ecosystems. For. Res. Pap..

[24] Arrese EL, Soulages JL (2010). Insect fat body: energy, metabolism, and regulation. Annu. Rev. Entomol..

[25] Sorge D, Nauen R, Range S, Hoffmann K (2000). Regulation of vitellogenesis in the fall armyworm, Spodoptera frugiperda (Lepidoptera: Noctuidae). J. Insect Physiol..

[26] Shu Y (2009). Molecular characterization and expression pattern of Spodoptera litura (Lepidoptera: Noctuidae) vitellogenin, and its response to lead stress. J. Insect Physiol..

[27] Rogers SL, Wiedemann U, Stuurman N, Vale RD (2003). Molecular requirements for actin-based lamella formation in Drosophila S2 cells. J. Cell Biol..

[28] Bustin SA (2009). The MIQE guidelines:Minimum Information for publication of quantitative real-time PCR experiments. Clin. Chem..

[29] Pfaffl MW (2001). A new mathematical model for relative quantification in real-time RT–PCR. Nucleic Acids Res..

