# Supplementary material for: Evaluation of Candidate Reference Genes for Quantitative Gene Expression Analysis in Spodoptera exigua after Long-time Exposure to Cadmium
Source: Sci Rep. 2017 Aug 21;7:8338. doi: 10.1038/s41598-017-08630-6 (PMC5567104; doi:10.1038/s41598-017-08630-6)
Supplement: Supplementary file 1 — Supplementary material [file 41598_2017_8630_MOESM1_ESM.pdf]

Evaluation of Candidate Reference Genes for Quantitative Gene Expression Analysis in *Spodoptera exigua* after Long-time Exposure to Cadmium.

Anna Płachetka-Bożek, Maria Augustyniak

Department of Animal Physiology and Ecotoxicology, University of Silesia in Katowice, Bankowa 9, PL 40-007 Katowice, Poland

Corresponding author E-mail: anna.plachetka@us.edu.pl (Anna Płachetka-Bożek)
Tel.: #48 32 3591196

Supplementary material

Captions

Figure S1. Total RNA from *Spodoptera exigua* measured using Agilent RNA 6000 Nano Kit.
Figure S2. Confirmation of primer specificity and amplicon size for housekeeping genes.

Table S1. Reference gene expression analysis with division into breeding strains and time-points.
Table S2. Reference gene expression analysis with and without division into breeding strains.
Table S3. Characteristics of gene-specific real-time PCR primers.

Figure S1. Total RNA from *Spodoptera exigua* measured using Agilent RNA 6000 Nano Kit.
Spodoptera, like Drosophila 28S rRNA, is processed into two fragments that migrate in a similar manner to the 18S rRNA (http://www.thermofisher.com/pl/en/home/references/ambion-tech-support/rna-isolation/general-articles/ribosomal-rna-sizes.html).
In this study, only perfect isolated probes that were visible in the wells: 1, 4-5 and 7-12, were used for mRNA expression analyzes.

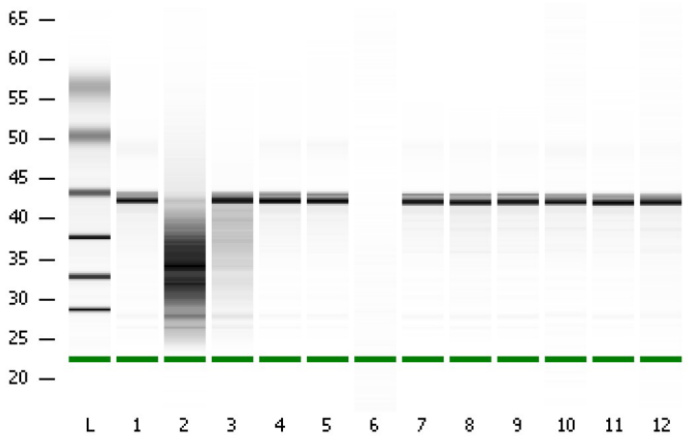

Figure S2. Confirmation of primer specificity and amplicon size for housekeeping genes.
Electrophoresis was performed on 2% agarose gel.

Abbreviations: ACT – cytoplasmic actin gene, TUBα – alpha tubulin, RpL7A – ribosomal protein L7A, RpL10 – ribosomal protein L10, EF2 – elongation factor 2, GAPDH – glyceraldehyde 3-phosphate dehydrogenase, G3PDH – glycerol-3-phosphate dehydrogenase.

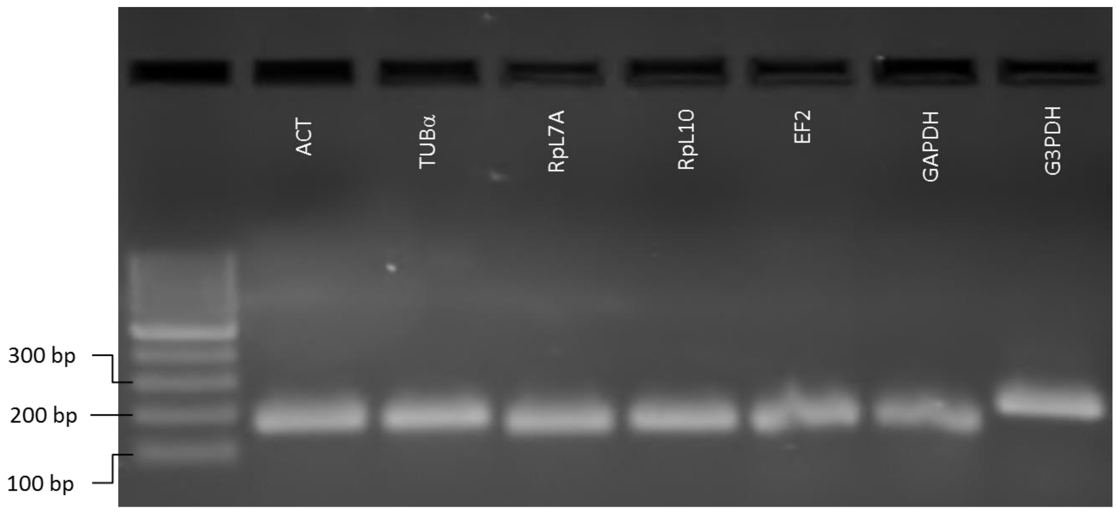

Table S1. Reference gene expression analysis with division into breeding strains and time-points.

Abbreviations: C – control strain, Cd – cadmium strain, 0h, 6h, 12h, 18h, 24h - time after eclosion, glyceraldehyde 3-phosphate dehydrogenase (GAPDH), glycerol-3-phosphate dehydrogenase (G3PDH), elongation factor 2 (EF2), ribosomal protein L7A (RpL7A), ribosomal protein L10 (RpL10), alpha tubulin (TUBα), cytoplasmic actin gene (ACT), average (AV), standard deviations (SD), median (ME), 25th and 75th quartiles (Q1 and Q3), minimum (Min) maximum (Max), odds (Odd) = Max-Min.

gene	*experimental group*	Av	SD	Me	Q1	Q3	Min	Max	Odd
GAPDH	*C 0h*	18.69	0.91	18.88	18.39	19.17	17.44	19.57	2.14
	Cd 0h	18.72	0.44	18.65	18.54	18.83	18.27	19.33	1.06
	C 6h	17.44	0.77	17.54	16.81	17.82	16.55	18.72	2.18
	Cd 6h	18.35	0.84	18.18	17.86	18.96	17.33	19.41	2.09
	C 12h	17.72	0.82	17.55	17.30	18.35	16.60	18.77	2.17
	Cd 12h	18.69	0.90	18.56	18.23	19.02	17.54	20.17	2.63
	C 18h	16.31	0.83	16.12	15.87	16.72	15.33	17.50	2.18
	Cd 18h	18.06	0.28	18.03	17.92	18.17	17.77	18.44	0.66
	C 24h	17.59	1.13	17.57	17.08	18.28	15.88	19.08	3.20
	Cd 24h	18.82	0.85	18.67	18.08	19.53	17.96	19.90	1.94
	C 30h	17.05	0.32	17.09	16.82	17.25	16.63	17.48	0.84
	Cd 30h	17.48	0.57	17.47	16.99	17.90	16.84	18.21	1.37
	C 36h	16.49	0.63	16.31	16.01	16.79	16.01	17.33	1.32
	Cd 36h	17.75	0.64	17.95	17.56	18.09	16.75	18.43	1.69
	C 42h	16.64	1.61	16.21	15.72	17.12	15.24	18.92	3.68
	Cd 42h	16.89	0.57	16.78	16.62	17.18	16.14	17.76	1.62
	C 48h	17.77	1.20	17.73	17.36	18.13	16.34	19.28	2.95
	Cd 48h	17.01	0.41	17.02	16.88	17.02	16.49	17.63	1.14
G3PDH	*C 0h*	21.04	0.85	21.11	20.40	21.75	20.15	21.79	1.64
	Cd 0h	21.48	0.61	21.38	21.04	21.82	20.93	22.26	1.34
	C 6h	20.74	0.48	20.68	20.57	20.95	19.99	21.50	1.51
	Cd 6h	21.45	0.46	21.74	21.08	21.78	20.83	21.81	0.98
	C 12h	21.04	0.73	20.89	20.51	21.45	20.28	22.17	1.89
	Cd 12h	21.99	0.68	21.87	21.44	22.43	21.31	22.99	1.68
	C 18h	19.74	0.55	19.68	19.53	20.16	18.89	20.41	1.52
	Cd 18h	21.15	0.29	21.09	20.94	21.31	20.90	21.53	0.63
	C 24h	20.93	0.81	21.20	20.36	21.61	19.74	21.67	1.94
	Cd 24h	21.96	0.88	22.05	21.50	22.67	20.61	22.88	2.27
	C 30h	19.96	0.38	19.93	19.82	20.10	19.38	20.60	1.22
	Cd 30h	20.21	0.43	20.29	20.11	20.49	19.46	20.64	1.18
	C 36h	19.79	0.47	19.87	19.49	20.17	19.23	20.20	0.97
	Cd 36h	20.48	0.92	20.63	19.85	20.90	19.33	21.68	2.35
	C 42h	19.77	0.36	19.92	19.64	19.97	19.35	20.03	0.67
	Cd 42h	20.20	0.56	20.07	19.79	20.40	19.68	21.16	1.48
	C 48h	21.14	1.45	21.06	20.30	21.89	19.51	22.93	3.42
	Cd 48h	19.80	0.21	19.74	19.71	19.84	19.57	20.14	0.57
EF2	*C 0h*	17.71	0.92	17.87	17.38	18.20	16.46	18.63	2.17
	Cd 0h	18.53	0.38	18.50	18.30	18.72	18.12	19.00	0.88
	C 6h	16.41	0.87	15.89	15.75	16.97	15.65	17.88	2.23
	Cd 6h	17.50	0.45	17.52	17.07	17.80	17.03	18.07	1.04
	C 12h	16.30	0.63	16.17	15.90	16.80	15.54	17.10	1.56
	Cd 12h	17.15	0.49	17.11	16.93	17.43	16.43	17.84	1.42
	C 18h	15.43	0.35	15.27	15.17	15.60	15.14	15.96	0.82
	Cd 18h	16.72	0.33	16.71	16.51	16.92	16.36	17.11	0.75
	C 24h	16.68	0.66	17.00	16.34	17.10	15.62	17.25	1.63
	Cd 24h	17.37	0.86	17.46	17.08	17.61	16.03	18.64	2.61
	C 30h	15.91	0.46	15.84	15.72	16.04	15.28	16.73	1.45
	Cd 30h	16.15	0.30	16.10	15.96	16.22	15.85	16.69	0.84
	C 36h	15.54	0.43	15.54	15.22	15.86	15.09	15.97	0.88
	Cd 36h	16.41	0.72	16.49	15.97	16.72	15.51	17.39	1.89
	C 42h	15.22	0.16	15.16	15.13	15.28	15.11	15.40	0.29
	Cd 42h	15.80	0.36	15.77	15.76	15.87	15.25	16.37	1.12
	C 48h	16.71	1.08	16.66	16.22	17.15	15.45	18.07	2.62
	Cd 48h	15.67	0.33	15.65	15.56	15.80	15.23	16.13	0.91
RpL10	*C 0h*	17.12	0.75	17.47	17.06	17.53	16.01	17.55	1.54
	Cd 0h	17.96	0.40	17.95	17.77	18.14	17.49	18.47	0.97
	C 6h	16.49	0.74	16.66	15.98	16.83	15.46	17.68	2.22
	Cd 6h	17.25	0.50	16.98	16.88	17.72	16.80	17.86	1.07
	C 12h	16.44	0.62	16.37	15.99	16.92	15.71	17.24	1.53
	Cd 12h	17.32	0.75	17.19	16.87	17.70	16.42	18.51	2.09
	C 18h	15.29	0.45	15.10	15.07	15.47	14.83	15.99	1.16
	Cd 18h	17.01	0.47	16.97	16.69	17.29	16.52	17.56	1.05
	C 24h	16.47	0.71	16.75	16.05	16.82	15.44	17.37	1.94
	Cd 24h	17.43	0.93	17.57	17.11	18.09	15.84	18.36	2.52
	C 30h	16.16	0.59	16.20	15.67	16.50	15.32	17.04	1.72
	Cd 30h	16.40	0.50	16.38	16.14	16.78	15.66	17.01	1.35
	C 36h	15.45	0.36	15.45	15.14	15.76	15.14	15.77	0.63
	Cd 36h	16.48	0.98	16.72	16.06	17.03	15.03	17.58	2.56
	C 42h	15.86	0.02	15.86	15.85	15.86	15.84	15.87	0.03
	Cd 42h	16.44	0.50	16.35	16.26	16.55	15.79	17.31	1.52
	C 48h	17.19	1.10	17.17	16.71	17.65	15.87	18.55	2.68
	Cd 48h	16.40	0.42	16.24	16.11	16.72	15.97	16.97	1.00

gene	*experimental group*	Av	SD	Me	Q1	Q3	Min	Max	Odd
	C 0h	17.99	0.87	18.09	17.58	18.49	16.88	18.92	2.04
	Cd 0h	18.86	0.53	18.89	18.60	19.15	18.20	19.46	1.27
	C 6h	17.46	0.85	17.78	16.84	17.88	16.25	18.74	2.49
	Cd 6h	18.05	0.53	17.74	17.64	18.61	17.63	18.65	1.02
	C 12h	17.41	0.73	17.16	16.86	17.92	16.71	18.47	1.76
	Cd 12h	18.12	0.71	18.07	17.74	18.60	17.13	19.05	1.92
	C 18h	16.42	0.60	16.20	15.96	16.62	15.96	17.39	1.43
	Cd 18h	17.89	0.47	17.86	17.67	18.08	17.35	18.48	1.13
**RpL7A**	*C 24h*	17.47	0.68	17.68	17.13	17.82	16.32	18.36	2.04
	Cd 24h	18.26	0.91	18.28	17.94	18.86	16.79	19.34	2.55
	C 30h	17.03	0.82	17.03	16.63	17.57	15.62	18.30	2.68
	Cd 30h	17.17	0.40	17.10	16.94	17.38	16.67	17.80	1.13
	C 36h	16.73	0.66	16.73	16.17	17.29	16.15	17.32	1.17
	Cd 36h	17.38	0.86	17.50	17.05	17.76	16.13	18.45	2.32
	C 42h	16.66	0.17	16.66	16.60	16.72	16.54	16.78	0.24
	Cd 42h	17.31	0.43	17.26	17.12	17.39	16.78	18.06	1.28
	C 48h	18.04	0.94	18.03	17.66	18.40	16.91	19.19	2.28
	Cd 48h	17.13	0.47	17.23	16.69	17.51	16.60	17.63	1.03
	C 0h	19.04	0.87	19.15	18.52	19.68	17.99	19.88	1.89
	Cd 0h	19.60	0.72	19.43	19.06	19.97	18.99	20.53	1.54
	C 6h	18.40	1.17	18.72	17.42	19.14	16.90	20.08	3.18
	Cd 6h	18.77	0.77	18.34	18.17	19.57	18.13	19.64	1.51
	C 12h	18.08	0.76	17.83	17.58	18.67	17.24	19.11	1.88
	Cd 12h	18.97	0.83	19.03	18.63	19.19	17.77	20.27	2.51
	C 18h	16.71	0.59	16.40	16.31	16.84	16.30	17.70	1.40
	Cd 18h	18.56	0.54	18.56	18.14	18.98	18.02	19.10	1.08
	C 24h	17.84	0.80	17.84	17.51	18.18	16.53	19.13	2.61
**TUBα**	*Cd 24h*	18.89	1.10	18.74	18.14	19.52	17.61	20.55	2.95
	C 30h	17.21	0.44	17.12	16.95	17.46	16.59	17.92	1.33
	Cd 30h	17.46	0.52	17.64	17.09	17.80	16.73	18.02	1.30
	C 36h	16.70	0.84	17.02	16.38	17.17	15.74	17.33	1.59
	Cd 36h	17.80	0.61	17.99	17.45	18.15	16.94	18.50	1.56
	C 42h	16.85	0.39	16.82	16.65	17.04	16.48	17.26	0.78
	Cd 42h	17.22	0.65	17.31	16.77	17.67	16.31	18.01	1.70
	C 48h	16.03	0.50	15.94	15.76	16.22	15.54	16.71	1.17
	Cd 48h	16.98	0.34	16.95	16.77	17.24	16.57	17.40	0.84
	C 0h	16.29	0.52	16.27	16.08	16.48	15.69	16.95	1.26
	Cd 0h	16.11	0.57	16.08	15.81	16.38	15.45	16.82	1.38
	C 6h	16.55	0.68	16.51	16.26	16.56	15.79	17.95	2.16
	Cd 6h	16.35	0.83	16.39	15.95	16.57	15.16	17.85	2.70
	C 12h	17.40	0.74	17.59	17.09	17.95	16.16	18.10	1.94
	Cd 12h	17.32	0.55	17.42	16.79	17.75	16.67	18.17	1.50
	C 18h	17.34	1.04	17.33	16.81	17.71	16.04	18.82	2.78
	Cd 18h	17.69	0.84	17.68	17.05	18.33	16.61	18.76	2.15
**ACT**	*C 24h*	18.36	0.71	18.11	17.84	18.81	17.59	19.50	1.91
	Cd 24h	18.51	1.04	18.37	18.08	18.78	16.59	20.30	3.71
	C 30h	18.00	0.31	17.97	17.74	18.22	17.64	18.47	0.84
	Cd 30h	18.05	0.63	17.94	17.79	18.32	17.23	19.45	2.22
	C 36h	17.82	0.35	17.85	17.67	18.00	17.38	18.21	0.82
	Cd 36h	17.71	1.11	17.92	17.49	17.95	15.67	19.61	3.94
	C 42h	18.58	0.26	18.59	18.45	18.71	18.32	18.83	0.51
	Cd 42h	18.32	0.55	18.34	17.78	18.78	17.69	19.09	1.41
	C 48h	18.91	0.08	18.89	18.86	18.94	18.85	19.03	0.18
	Cd 48h	18.27	0.69	18.56	18.32	18.65	16.91	18.70	1.80

Table S2. Reference gene expression analysis with and without division into breeding strains.

Abbreviations: glyceraldehyde 3-phosphate dehydrogenase (GAPDH), glycerol-3-phosphate dehydrogenase (G3PDH), elongation factor 2 (EF2), ribosomal protein L7A (RpL7A), ribosomal protein L10 (RpL10), alpha tubulin (TUBα), cytoplasmic actin gene (ACT), average (AV), standard deviations (SD), median (ME), 25th and 75th quartiles (Q1 and Q3), minimum (Min) maximum (Max), odds (Odd) = Max-Min.

gene	*experimental group*	AV	SD	Me	Q1	Q3	Min	Max	Odd
**GAPDH**	*Control*	17.26	1.06	17.27	16.61	17.73	15.24	19.57	4.34
	Cadmium	17.95	0.94	17.96	17.23	18.43	16.14	20.17	4.03
	All samples	17.63	1.05	17.59	16.85	18.39	15.24	20.17	4.93
**G3PDH**	*Control*	20.43	0.80	20.28	19.89	20.89	18.89	22.17	3.28
	Cadmium	20.96	0.98	20.93	20.13	21.71	19.33	22.99	3.67
	All samples	20.73	0.95	20.61	19.97	21.52	18.89	22.99	4.10
**EF2**	*Control*	16.20	0.89	15.87	15.61	16.94	15.09	18.63	3.54
	Cadmium	16.76	0.96	16.69	15.95	17.45	15.23	19.00	3.77
	All samples	16.50	0.96	16.30	15.78	17.09	15.09	19.00	3.91
**RpL10**	*Control*	16.21	0.77	16.05	15.66	16.79	14.83	17.68	2.85
	Cadmium	16.94	0.80	16.89	16.35	17.53	15.03	18.51	3.48
	All samples	16.68	0.85	16.72	16.01	17.24	14.83	18.55	3.72
**RpL7A**	*Control*	17.20	0.82	17.09	16.66	17.80	15.62	18.92	3.30
	Cadmium	17.76	0.79	17.72	17.18	18.40	16.13	19.46	3.34
	All samples	17.52	0.85	17.50	16.89	18.03	15.62	19.46	3.85
**TUBα**	*Control*	17.53	1.11	17.45	16.82	18.03	15.54	20.08	4.54
	Cadmium	18.21	1.08	18.02	17.44	18.96	16.31	20.55	4.24
	All samples	17.87	1.14	17.72	16.96	18.71	15.54	20.55	5.01
**ACT**	*Control*	17.55	0.94	17.73	16.75	18.13	15.69	19.50	3.81
	Cadmium	17.64	0.87	17.84	16.85	18.28	15.16	20.30	5.14
	All samples	17.69	1.02	17.84	16.94	18.39	15.16	20.30	5.14

Table S3. Characteristics of gene-specific real-time PCR primers.
Primer sequence, amplification length, amplicon sequence, qPCR efficiency of the primers used for the potential reference genes.

<table>
<tr><th>ACT</th><th>gi|40218737|gb|AY507963.1|</th></tr>
<tr><td>Primer forward</td><td>TGCGTGACATCAAGGAGAAG</td></tr>
<tr><td>Primer reverse</td><td>AG AAG GAA GGC TGG AAG AGG</td></tr>
<tr><td>Amplicon size</td><td>174 bp</td></tr>
<tr><td>Amplicon sequence</td><td>TGCGTGACATCAAGGAGAAGCTGTGCTATGTCGCCCTCGACTTCGAGCAGGAGATGGCCACCGCTGCCGCCTCCACCTC
CCTCGAGAAGTCCTACGAACTTCCCGACGGTCAGGTCATCACCATCGGTAACGAGAGGTTCCGTTGCCCTGAAGCCCTCT
TCCAGCCTTCCTTCT</td></tr>
<tr><td>Efficiency</td><td>90.7%</td></tr>
<tr><th>GAPDH</th><th>gi|33’283895|gb|JF728815.1|</th></tr>
<tr><td>Primer forward</td><td>CTGAGGAACAGGTCGTGTCATCCGA</td></tr>
<tr><td>Primer reverse</td><td>GATCGATAACGCGGTTGGAGTAGCC</td></tr>
<tr><td>Amplicon size</td><td>150 bp</td></tr>
<tr><td>Amplicon sequence</td><td>CTGAGGAACAGGTCGTGTCATCCGATTTCATTGGTGACAACCACTCATCTATCTTCGATGCTGCTGCCGGTATCTCTCTG
AACGACAACTTCGTCAAGCTCATCAGCTGGTATGACAACGAGTTTGGCTACTCCAACCGCGTTATCGATC</td></tr>
<tr><td>Efficiency</td><td>95.45%</td></tr>
<tr><td>Citation</td><td>Zhu et al., 2014</td></tr>
<tr><th>G3PDH</th><th>gi|544196752|gb|KF170736.1|</th></tr>
<tr><td>Primer forward</td><td>GTAGTTCCCCACCAGTTTGTCAG</td></tr>
<tr><td>Primer reverse</td><td>CACCTCAGACGCAATGTTAGC</td></tr>
<tr><td>Amplicon size</td><td>198 bp</td></tr>
<tr><td>Amplicon sequence</td><td>GTAGTTCCCCACCAGTTTGTCAGAACTATTTGTTCTACACTCCTCGGGAAGATAAAGCCGACCGCAGCTGCACTTTCTTTG
ATAAAGGGCTTCGATATCGCGGAAGGCGGTGGCATTGACCTCATTTCTCACATTATTACAAGGTGCCTCAAAATCCCATG
TGCCGTATTAATGGGAGCTAACATTGCGTCTGAGGTG</td></tr>
<tr><td>Efficiency</td><td>94.65%</td></tr>
<tr><td>Citation</td><td>Teng et al., 2011</td></tr>
<tr><th>EF2</th><th>gi|28627568|gb|AY078407.1|</th></tr>
<tr><td>Primer forward</td><td>CTGACCGCGCAACCCAGACT</td></tr>
<tr><td>Primer reverse</td><td>CACGAACATGGGGGTACCAGCG 3</td></tr>
<tr><td>Amplicon size</td><td>150 bp</td></tr>
<tr><td>Amplicon sequence</td><td>CTGACCGCGCAACCCAGACTTATGGAGCCCGTATACTTGTGCGAGATTCAGTGTCCTGAGGTCGCCGTCGGTGGTATCT
ACGGTGTACTGAACAGACGTCGTGGTCACGTATTCGAGGAGTCTCAGGTCGCTGGTACCCCCATGTTCGTG</td></tr>
<tr><td>Efficiency</td><td>91.8%</td></tr>
<tr><td>Citation</td><td>Zhu et al., 2014</td></tr>
<tr><th>RpL10</th><th>gi|160947857|gb|EU258622.1|</th></tr>
<tr><td>Primer forward</td><td>GGCTACGGTCGACGACTTCCC</td></tr>
<tr><td>Primer reverse</td><td>GCAGCCTCATGCGGATGTGGAAC</td></tr>
<tr><td>Amplicon size</td><td>155 bp</td></tr>
<tr><td>Amplicon sequence</td><td>GGCTACGGTCGACGACTTCCCACTATGCGTCCACTTGGTATCTGACGAATACGAGCAGCTTAGCTCTGAAGCTCTGGAA
GCAGGCCGTATTTGCTGCAACAAGTACCTTGTTAAGAACTGCGGCAAAGATCAGTTCCACATCCGCATGAGGCTGC</td></tr>
<tr><td>Efficiency</td><td>91.8%</td></tr>
<tr><td>Citation</td><td>Zhu et al., 2014</td></tr>
<tr><th>RpL7A</th><th>gi|161015754|gb|EU25’814.1|</th></tr>
<tr><td>Primer forward</td><td>TGAGCTTGTCCTCTTCCTGCCC</td></tr>
<tr><td>Primer reverse</td><td>GCTGCACGGTCGCCAGACTC</td></tr>
<tr><td>Amplicon size</td><td>150 bp</td></tr>
<tr><td>Amplicon sequence</td><td>TGAGCTTGTCCTCTTCCTGCCCGCCCTTTGCCGTAAAATGGGCGTGCCCTACTGTATTGTCAAGGGCAAATCCCGCCTGG
GAGCCCTTGTACACCGCAAGACTTGCACCTGCGTAGCAATCACACATGTTGAGTCTGGCGACCGTGCAGC</td></tr>
<tr><td>Efficiency</td><td>94.7%</td></tr>
<tr><th>TUBα</th><th>gi|302403434|gb|GU983’15.1</th></tr>
<tr><td>Primer forward</td><td>CGTGACGACGTGTCTGCGGT</td></tr>
<tr><td>Primer reverse</td><td>GCGTGAGCTCGGGTACGGTG</td></tr>
<tr><td>Amplicon size</td><td>167 bp</td></tr>
<tr><td>Amplicon sequence</td><td>CGTGACGACGTGTCTGCGGTTCCCTGGTCAGCTGAATGCGGATCTCCGCAAGCTGGCAGTCAACATGGTGCCGTTCCCG
CGTCTCCACTTCTTCATGCCCGGTTTCGCTCCCTTGACATCTCGCGGCAGCCAGCAATACCGCGCCCTCACCGTACCCGAG
CTCACGC</td></tr>
<tr><td>Efficiency</td><td>93.3%</td></tr>
<tr><td>Citation</td><td>Zhu et al., 2014</td></tr>
</table>
